# Risk of peripheral artery disease and stroke in migraineurs with or without aura: a nationwide population-based cohort study

**DOI:** 10.7150/ijms.72119

**Published:** 2022-07-04

**Authors:** Wun-Zhih Siao, Chun-Hung Su, Yu-Hsiang Kuan, Tung-Han Tsai, Kuang-Hua Huan, Chien-Ying Lee

**Affiliations:** 1 Department of Internal Medicine, School of Medicine, Chung Shan Medical University, Taichung, Taiwan; 2 Division of Cardiology, Department of Internal Medicine, Chung Shan Medical University Hospital, Taichung, Taiwan; 3 Institute of Medicine, College of Medicine, Chung Shan Medical University, Taichung, Taiwan; 4 Department of Pharmacology, School of Medicine, Chung Shan Medical University, Taichung, Taiwan; 5 Department of Pharmacy, Chung Shan Medical University Hospital, Taichung, Taiwan; 6 Department of Health Services Administration, China Medical University, Taichung, Taiwan

## Abstract

**Background:** Migraine is deemed a neurovascular disorder and there is growing evidence on the increased risk of cardiovascular disease, especially ischemic stroke, in patients with migraine. However the risk of peripheral artery disease (PAD) and stroke in migraineurs and the association between migraineurs with or without aura is still under debate. Our study aimed to identify the risk of PAD and stroke in migraineurs with or without aura.

**Methods:** This was a population-based cohort study utilizing Taiwan Longitudinal Health Insurance Database (LHID2010). Patients with coding of migraine from 2002 to 2011 were enrolled and those with established cardiovascular disease defined as myocardial infarction, stroke, PAD, venous thromboembolism, atrial fibrillation and heart failure diagnosis before the index date were excluded. Participants were categorized into migraine group, migraine without aura group, and migraine with aura group respectively. The subjects in the three groups were propensity score-matched randomly to their counterparts without migraine. The study outcome was PAD and stroke. The Cox proportional hazard model was used to estimate the hazard ratios with 95% confidence interval (CI) for the association between migraine and the incident events of disease, after controlling for related variables.

**Results:** The migraine, migraine without aura, and migraine with aura group included 5,173 patients, 942 patients and 479 patients respectively after propensity score-matching. The migraine group had an increased risk of PAD [adjusted hazard ratio (aHR): 1.93; 95% confidence interval (CI): 1.45-2.57; p < 0.001] and stroke (aHR: 1.55; 95% CI: 1.35-1.77; p < 0.001) compared to their non-migraine controls. Both the groups of migraine without aura and with aura had an increased risk of stroke (aHR: 1.49, 95% CI: 1.11-2.00; p = 0.008; aHR: 1.63, 95% CI: 1.10-2.43; p = 0.016). With regards to the outcome of PAD, the group of migraine with aura had a trend of an increased risk but did not reach statistical significance (aHR: 1.95, 95% CI: 0.86-4.40; p = 0.108).

**Conclusion:** Migraineurs without established cardiovascular disease had a significantly increased risk of PAD and stroke, and the risk of stroke persists in migraineurs with or without aura, with an increased trend of PAD in migraineurs with aura. Our study result should remind clinical physicians of the risk of PAD in the future among migraineurs even without established cardiovascular disease currently, and screening for PAD and stroke may be needed in caring patients with migraine.

## Introduction

Migraine is deemed as a neurovascular disorder that is more common in women than men 1. A population-based study showed high prevalence of migraine in Taiwan, with prevalence rate of 14.4% in woman and 9.1% in men aged more than 15 years old respectively 2. Migraine can lead to disability, impair life quality, and carry large healthcare and economic burden. The association between migraine and cardiovascular disease has been studied. Strong evidence suggested migraine as a risk factor for ischemic stroke. Researches have also shown the positive link between migraine and ischemic heart disease, especially in migraineurs with aura 3-5.

PAD has approximately 20% prevalence rate and the risk increased in elderly. Atherosclerosis is the major pathogenesis that implicated in the development of PAD. Risk factors such as cigarette smoking, diabetes, hypertension, dyslipidemia, and hyperhomocysteinemia contribute to the development of PAD 6, 7. Severe PAD increased the risk of limb amputation or percutaneous transluminal intervention that result in poor life quality and heavy burden of economic. Once patients with PAD develop critical limb ischemia, the mortality rate within the first year was as higher as 20-25%, and the five-year survival rate was less then 30% 8. There was substantial evidence supporting the higher risk of stroke among migraineurs, and mostly migraineurs with aura 5. Historical researches demonstrated undetermined results as regard the association of migraine and PAD. Evidence from observation studies found migraineurs had lower ankle-brachial index (ABI) or higher risk with symptom of claudication suggesting presence of PAD 9, 10. However a population-based cohort study did not showed a statistically significant correlation between migraine and PAD 11. Moreover, there is little data addressing the risk of PAD in migraineurs with or without aura. Thus the aim of this study was to evaluate the risk of PAD and stroke in migraineurs without established cardiovascular disease, and the risk between migraineurs with or without aura, by analyzing a nationwide population-based retrospective cohort from the National Health Insurance Research Database (NHIRD) in Taiwan.

## Materials and Methods

### Database

This was a retrospective cohort study, in which secondary data analysis was conducted. Data were obtained from the 2000-2013 Longitudinal Health Insurance Database (LHID) provided by the National Health Insurance Administration, Ministry of Health and Welfare and managed by National Health Research Institutes (Registered number NHIRD-104-004). The LHID comprises the information of one million beneficiaries randomly selected from the Taiwan National Health Insurance (NHI) program. The NHI program is a nationwide social insurance program that has covered up to 99% of citizens since 1995. Hence, the database is nationally representative of Taiwan. Owing to the anonymity of the database, the requirement for informed consent was waived, and this study was approved as an ethical review by the Institutional Review Board of China Medical University Hospital, Taiwan.

### Study Subjects

The participants were the new-onset migraine patients who aged above 20 years old. The definition of migraine was migraine diagnosis three times a year, which according to the International Classification of Diseases, Ninth Revision, Clinical Modification (ICD-9-CM) code 346), and concurrent use of antimigraine preparations. We excluded patients who had suffered cardiovascular diseases before had migraine to reduce the study bias. The cardiovascular diseases contained myocardial infarction (ICD-9-CM code 410), stroke (ICD-9-CM code 430-438), peripheral artery disease (ICD-9-CM) code 443.9), venous thromboembolism (ICD-9-CM code 453), atrial fibrillation (ICD-9-CM code 427.31), and heart failure (ICD-9-CM code 428). Furthermore, we used the propensity score matching (PSM) to obtain the patients without migraine in 1:5 matching for each migraine patient on the year of enrollment, via gender, age and Charlson comorbidities index (CCI), as the comparison. In addition, the patients who we selected for the comparison was had no received any migraine diagnosis during the study period. The study is an epidemiologic study based on the LHID 12, 13. We adopted the PSM to obtain the comparison group the same method as previous population-based studies conducted on LHID. The PSM is a statistical matching technique that is available to reduce potential confounding caused by unbalanced covariates in non-experimental settings. The PSM is the probability calculated via the Logistic regression model. A score is a unit with certain characteristics that will be assigned to migraine patients. The scores could be used to reduce or eliminate selection bias in observational studies on the characteristics of migraine and non-migraine.

**Figure 1:**
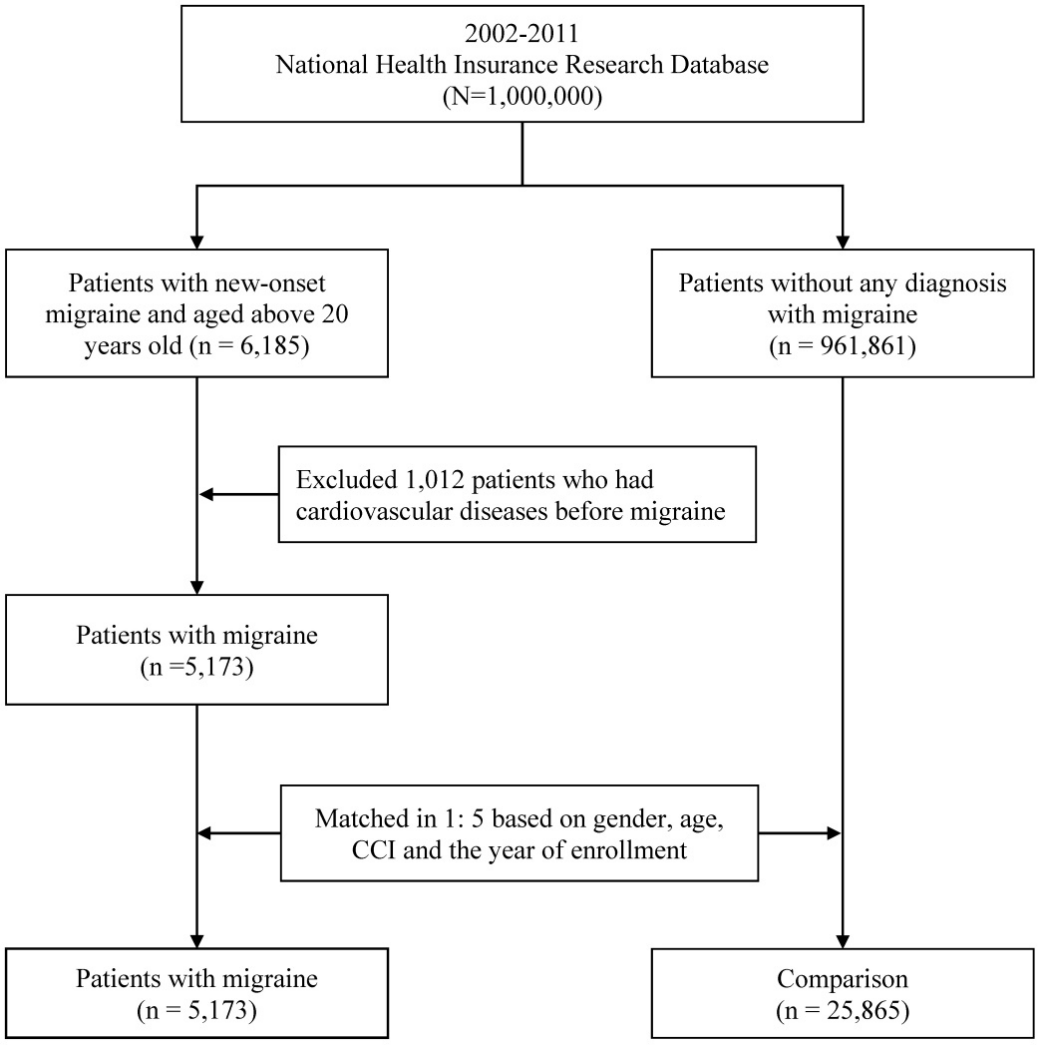
Flowchart of the study subject selection process.

We enrolled 5,173 patients with migraine and 25,865 patients without migraine, respectively. Besides, we classified subgroups of migraine patients into migraine without aura (ICD-9 CM code 346.1, 346.7), and migraine with aura (ICD-9 CM code 346.0, 346.5, 346.6). Figure 1 was the flowchart of selection of patients.

### Study Designs

This study was conducted using 2000-2013 claim data from LHID. We enrolled patients with migraine and the comparison from 2002-2011 as the study subject, to ensure that exclusion condition and each patient had least two years of follow-up. Each patient was follow-up until the date of the incident events of disease, death, or the end of 2013, whichever occurred first. We estimated the risk of the incident events of disease contained peripheral artery disease and stroke. The definition of peripheral artery disease in the study was based on the diagnosis according to the ICD-9-CM codes 443.9, and the stroke was according to the ICD-9-CM codes 430-438.

The control variables in the study were gender, age, and comorbidity diseases. The comorbidity disease contained diabetes mellitus (ICD-9-CM codes 250), hypertension (ICD-9-CM codes 401-405), hyperlipidemia (ICD-9-CM code 272.0-272.4), valvular heart disease (ICD-9-CM code 394-396, 424, 746), obesity (ICD-9-CM code 278), chronic obstructive pulmonary disease (COPD) (ICD-9-CM code 490-492, 494-496), liver disease (ICD-9-CM code 070, 571, 573.3), cancer (ICD-9-CM code 140-239), thyroid disease (ICD-9-CM code 240-246, 759.1-759.2), autoimmune disease (ICD-9-CM code 279.4), renal failure (ICD-9-CM code 584-585), and Helicobacter pylori (ICD-9-CM code 041.86).

### Statistical analysis

All statistical analyses in the study were using SAS software version 9.4 (SAS Institute Inc., Cary, NC, USA). Descriptive statistics were used to perform descriptive analysis of the number and percentage of patient characteristics (gender, age, and comorbidity disease). The Chi-square test was used to compare the differences between migraine patients and the comparison after matching. The study used the Cox proportional hazard model to estimate the hazard ratios with 95% confidence interval (CI) for the association between migraine and the incident events of disease, after controlling for related variables. Statistical significance in this study was defined as p < 0.05.

## Results

### The baseline characteristics of patients with migraine after matching

Table 1 show the distribution of the variables of the study subjects. Among all patients with migraine, there were 942 patients without aura (3.03%), and 479 patients with aura (1.54%). In patients with migraine, the age was 44.97 ± 13.36, and most patients were female (72.82%). In terms of comorbidities, 8.93% of patients with migraine had diabetes mellitus, 28.44% had hypertension, 18.25% had hyperlipidemia, 4.99% had valvular heart disease, and 24.03% had liver disease. the gender, age and CCI were the matched and the Chi-square test was used to verify the difference between the paired migraine patients and the comparison, which there was no statistically significant difference (p >0.05).

### Risk of related health outcomes in migraine patients with or without aura

**Figure 2:**
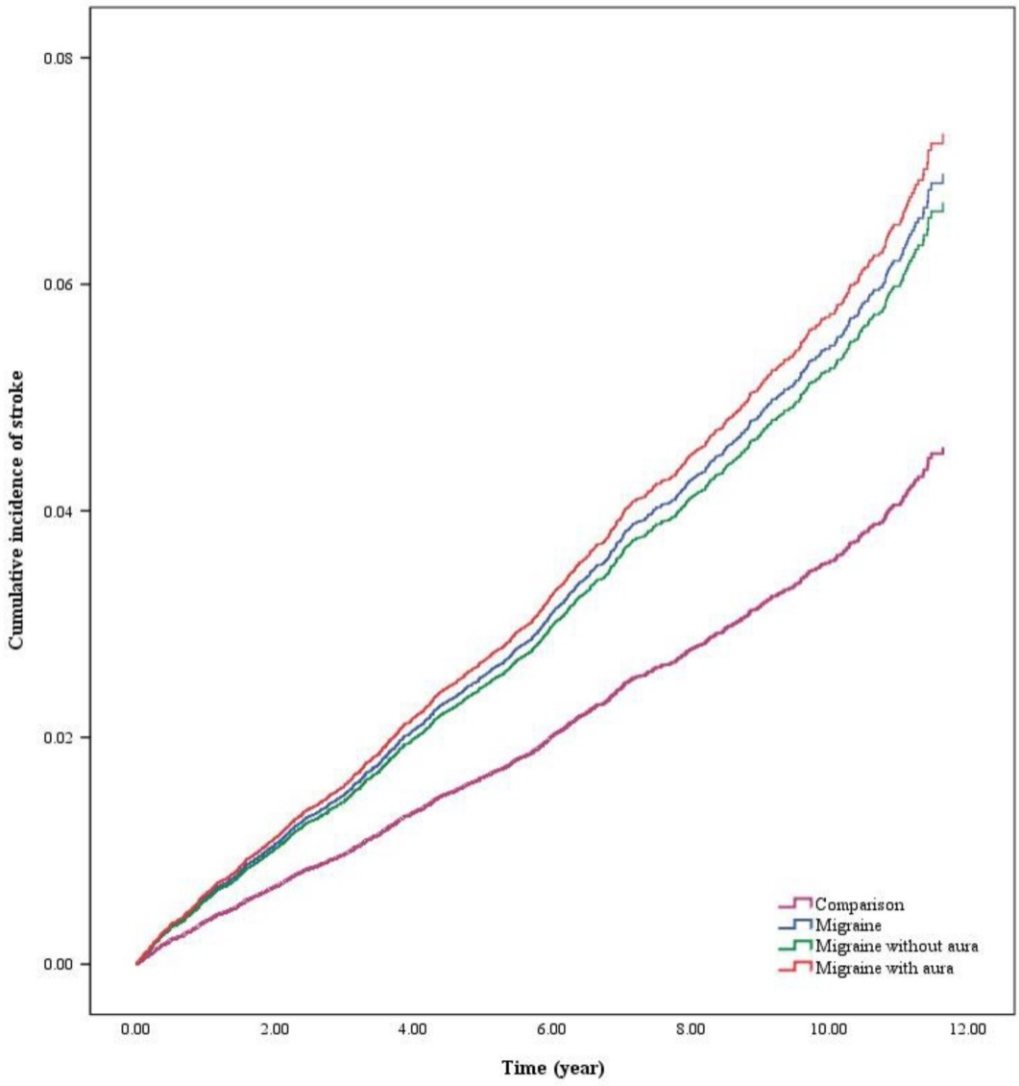
The cumulative risk of stroke in migraine patients with or without aura.

**Figure 3:**
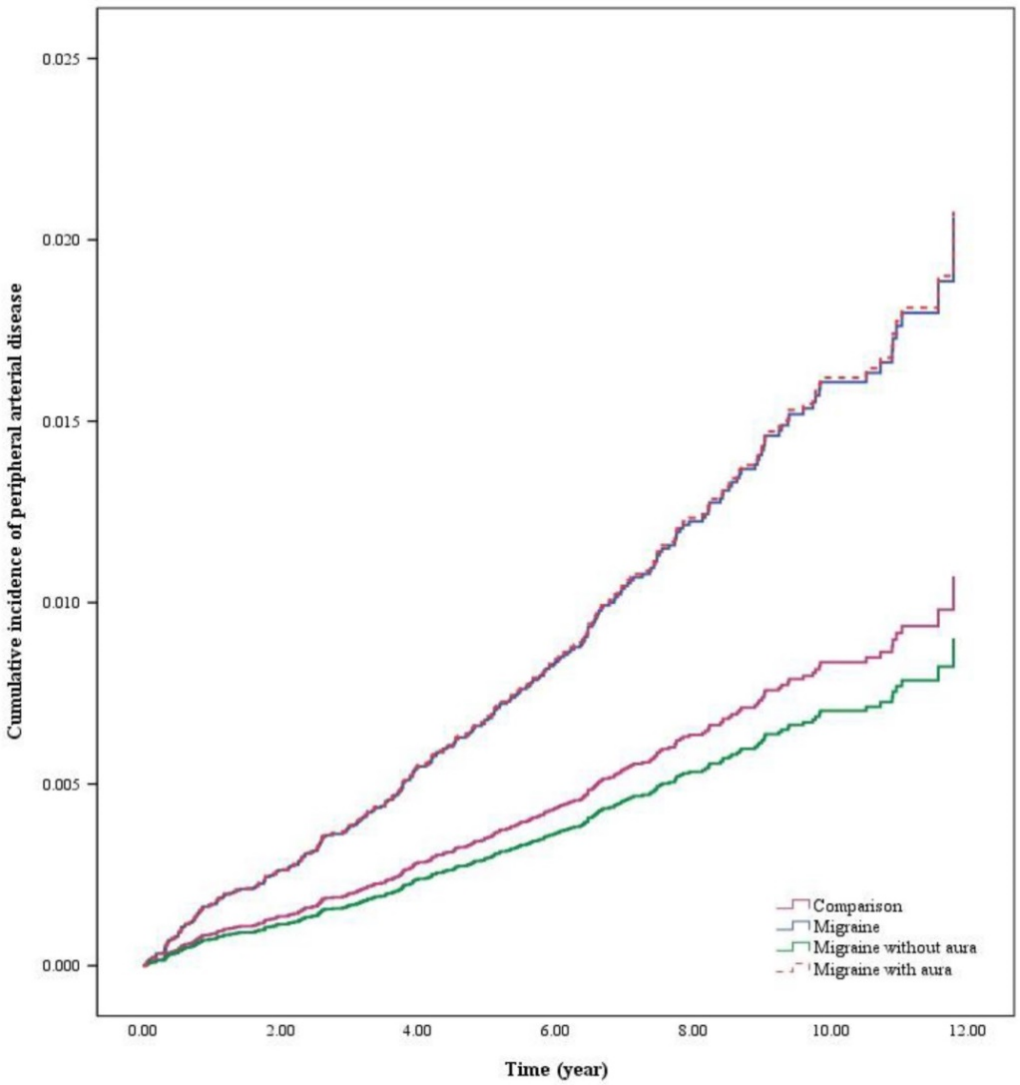
The cumulative risk of peripheral artery disease in migraine patients with or without aura.

This study explores the correlation between migraine patients and the occurrence of peripheral artery disease and stroke. Figure 2 illustrated the cumulative risk of stroke was significantly higher in migraine patients than in the comparison. As well, Figure 3 illustrated the cumulative risk of peripheral artery disease was higher in migraine patients than in the comparison.

Table 2 shows the risk of each variable and the occurrence of peripheral artery disease and stroke, respectively. After controlling other related variables, the adjusted hazard ratio (aHR) of migraine patients in developing peripheral artery disease was 1.93 times (95 % CI =1.45-2.57), and stroke was 1.55 times (95% CI=1.35-1.77). In terms of subgroup, migraine without aura and with aura patients had no statistic significant on developing peripheral artery disease, but migraine without aura patients (aHR =1.49, 95% CI =1.11-2.00) and migraine with aura patients (aHR =1.63, 95% CI =1.10-2.43) both had a higher risk on developing stroke. The more old age, the higher the relative risk of developing peripheral artery disease and stroke. Compared with patients aged 20-34, the relative risk of patients in ≥65 years old was 8.37 times (95% CI =4.62-15.18) on developing peripheral artery disease and 17.81 times (95% CI =13.19-24.06) on developing stroke.

In terms of comorbidity analysis, patients with hypertension showed the higher relative risk of developing peripheral artery disease (aHR=1.65, 95% CI=1.24-2.19); patients with diabetes mellitus (aHR=1.29, 95% CI=1.13-1.48), hypertension (aHR=1.65, 95% CI=1.46-1.87), and valvular heart disease (aHR= 1.42, 95% CI=1.15-1.76) had the higher risk of developing stroke.

## Discussion

Our study showed migraineurs without established cardiovascular disease had a 1.93-fold risk increment of PAD. The increased risk of stroke in migraineurs was also observed (aHR: 1.55; 95% CI: 1.35-1.77; p < 0.001), which was compatible with the data from meta-analysis study (aHR: 1.73; 95% CI: 1.31-2.29; p : 0.004) 14. Evidence had showed migraineurs had higher risk of ischemic stroke particularly those with aura 14, 15. Our study revealed both migraineurs with or without aura had increased risk of stroke, with 1.63-fold risk increment and 1.49-fold risk increment respectively. Although no statistical significant association between PAD and migraineurs with aura was found, the increased trend of risk of PAD in migraineurs with aura was observed in our study.

There are several possible mechanisms link between migraine and PAD. Endothelial microparticles (EMP) are small vesicles generated from endothelial cells and play important roles in inflammation, coagulation and endothelial function. Elevated EMP was observed among woman with migraine and aura, and was correlated with higher heart rate-adjusted augmentation index, which is an indirect measure of arterial stiffness 16. The vascular biomarker that implicated in the pathogenesis of coagulation-specific disorder has also contributed the association of migraine and PAD. A population-based study showed woman with migraine and aura had elevated Fibrinogen and Factor II level, and the increased frequency of aura attack predicted higher vascular biomarker 17. The molecular basis may provide support of our result that increased risk of PAD in migraineurs and especially those with aura.

Accumulating evidence also suggested the genetic overlaping between migraine and coronary artery disease 18. Since potential pathophysiology that promote atherosclerotic cardiovascular disease is involved in the shared genetic factors between coronary artery disease and PAD 19, the investigation of genetic basis may provide our understanding on the contributing mechanism between the two entities of disease.

Selvin, Elizabeth and Thomas P. Erlinger had described diabetes, hypertension, hypercholesterolemia, and impaired renal function as the risk factors of PAD 20. In our study, participants with history of diabetes, hypertension and renal failure were positive associated with PAD, which was compatible the result of the previous study. Subjects with chronic obstructive pulmonary disease and hypercholesterolemia also have higher risk of PAD albeit non-statistical significance. Of note, the obesity paradox has been mentioned 21 and Selvin, Elizabeth and Thomas P. Erlinger showed obesity was negative associated with PAD 20. Our study also demonstrated the trend of negative association between PAD and obesity.

Previous study had suggested the strong evidence of positive correlation between migraine and stroke. A case-control study demonstrated the association between migraine and stroke (OR 1.5, 95% CI 1.2-2.1), and claudication (OR 2.69, 95% CI 1.98-3.23) 10. Of note, the study population did not exclude those with established cardiovascular disease. Evidence from meta-analysis study found the higher risk of ischemic stroke among patients with migraine and the risk was increased by being female gender and younger age 14. The result from the meta-analysis disclosed the underlying pathophysiology beyond atherosclerosis between migraine and stroke, since atherosclerosis is classed as a disease of aging.

The historical studies aimed to investigate the relationship between migraine and PAD but the result was conflicting. A small study demonstrated that migraineurs tended to have lower ABI significantly. More specifically, participants in this study had no history of cardiac vascular events such as angina, heart disease or stroke 9. A cross-sectional case-control study was designed to identify the risk of atherosclerosis in migraineurs. Atherosclerosis was quantified by intima media thickness, pulse wave velocity and ABI. The result showed the risk of atherosclerosis did not increase among those with migraine compared with those without migraine, although subjects with migraine are more likely to have diabetes and smoking 22.

A population-based cohort study demonstrated the increased risk of PAD among subjects with migraine 23. In the study, patients with cardiovascular disease or cardiovascular risk were included. The bias of underlying cardiovascular disease attributed to the positive result cannot be ignored, since both cardiovascular disease and PAD shared pathogenic mechanisms. Otherwise there were still potential residues confounding bias such as autoimmune disease in the study. The autoimmune disease shared an inflammatory process that lead to development of atherosclerosis contributing to PAD risk 24. A retrospective study also conducted that rheumatoid arthritis as a significant risk factor of PAD 25. In our study, we excluded those with established cardiovascular disease to attenuate the influence of atherosclerosis. Otherwise, obesity and autoimmune disease were matched between the study groups to eliminate the effect of confounding bias. Another population-based cohort study showed the trend of lower incidence of peripheral artery disease in migraineurs but did not reach statistical significance. In the study, migraineurs with aura seems to have higher risk of peripheral artery disease compared with those without aura. The reasons for insignificant result maybe explain by the fewer events 11.

Otherwise, the frequency of infections may link the association between migraine and PAD. A study has yielded the increased likelihood of peripheral arterial disease when exposure more infection 26. A case-control study has also described the positive relationship between infections, such as Helicobacter pylori (*H. pylori*), and PAD, with odds ratio from 1.6-2.0 in young woman, and the risk is correlated with the cumulative number of infections 27. The clinical evidence also supported *H. pylori* to be a potential risk factor of PAD 28, 29. On the other hand, *H. pylori* infection was common in those with migraine, and eradication therapy lead to improved symptom of migraine 30, 31. During the era of the coronavirus disease 2019 (COVID-19) pandemic, headache was reported as a symptom of COVID-19 illness. Observation study has proposed the possible pathophysiology linked between COVID-19 infection and migraine, included calcitonin gene-related peptide, angiotensin system, inflammatory cytokines and trigeminovascular activation 32. In addition, patients with COVID-19 are at increased risk of PAD, even acute limb ischemia, particular those with higher inflammatory markers 33, 34. Inflammation may play a role linking migraine and PAD and more evidence needed to investigate the association.

PAD has potential risk to carry impairment in the quality of life and the prevalence rate is higher in woman at young age. Our study found increased risk of PAD in those with migraine and it's important to early screen the presence of PAD among migraineurs especially with aura, since the disease onset of migraine starting during young onset, and the underdiagnose of PAD may contribute to worse outcome 35, 36.

There are some strengths in our study. We examine the risk of PAD in migraineurs with and without aura, and the group of migraine with aura tended to have higher risk of PAD, albeit non-statistical significance which was related to limited events. As regard of the other study outcome of stroke, our study showed the same result of relationship between stroke and migraine, which makes our study more convincing. Our study also aimed to eliminate the possible confounding bias as much as possible and excluded those with underlying cardiovascular disease to attenuate the effect of atherosclerosis.

We highlight the following limitations in our study. First, the study was designed to attenuate the effect of established cardiovascular disease on the development of PAD among migraineurs. However, our study did not exclude migraineurs with cardiovascular risk such hypertension or diabetes, due to the limitation of effective sample size. However, the study groups were propensity score-matched by cardiovascular risk and Charlson Comorbidity Index to eliminate the possible confounding bias. Second, the drug effect, such as beta-blocker and ergot which may be used for treatment of migraine, may influence the result of our study on the risk of PAD in migraineurs. Third, the other residual biases still exist, such as the experience of infection frequency or inflammation biomarkers that are not available from database, which may be associated with risk factors for PAD. Fourth, among all migraine patients, a portion of patients were not identified as migraine with or without aura, due to the ICD code of the NHIRD. It may be an underestimation of our study regarding the risk of PAD among migraineurs with/without aura since there were some migraineurs, especially those without aura, not classified into their corresponding groups. Finally, we did not include cigarette smoking as a covariate because the NHIRD does not contain information on the quality of life, education, and living habits. However, we aimed to attenuate the bias of smoking between study and control groups by selecting chronic obstructive pulmonary disease (COPD) as the covariant.

## Conclusion

In summary, migraineurs without established cardiovascular disease had a significant increased risk of PAD and stroke, and the risk of stroke persists among migraineurs with or without aura, albeit the trend is toward higher likelihood of PAD in migraineurs with aura, but the result did not reach statistical significance. Our study result should remind clinical physicians the risk of PAD in the future among migraineurs even without established cardiovascular disease currently. More, screening for peripheral artery disease may be needed in caring for patients with migraine.

## Supplementary Material

Supplementary table.

## Figures and Tables

**Table 1:** The baseline characteristics of patients with migraine after matching

Variables	Comparison		Migraine patients								p-value ^b^
			subtotal		Migraine		Without aura		With aura		
	n	%	n	%	n	%	n	%	n	%	
Total	25,865	83.33	5,173	16.67	3,752	12.09	942	3.03	479	1.54	
Gender ^a.^											1.000
Female	18,835	72.82	3,767	72.82	2,697	71.88	708	75.16	362	75.57	
Male	7,030	27.18	1,406	27.18	1,055	28.12	234	24.84	117	24.43	
Age (year)^ a.^	45.00 ± 14.38		44.97 ± 13.36		45.64 ± 13.46		43.13 ± 12.98		43.32 ± 12.93		1.000
20-34	6,545	25.30	1,309	25.30	887	23.64	282	29.94	140	29.23	
35-49	10,700	41.37	2,140	41.37	1,555	41.44	379	40.23	206	43.01	
50-64	6,395	24.72	1,279	24.72	956	25.48	223	23.67	100	20.88	
≥65	2,225	8.60	445	8.60	354	9.43	58	6.16	33	6.89	
CCI^ a.^											1.000
0	12,025	46.49	2,405	46.49	1,734	46.22	455	48.30	216	45.09	
1	7,535	29.13	1,507	29.13	1,071	28.54	279	29.62	157	32.78	
2	3,265	12.62	653	12.62	488	13.01	110	11.68	55	11.48	
≥3	3,040	11.75	608	11.75	459	12.23	98	10.40	51	10.65	
Diabetes mellitus											<0.001
No	22,923	88.63	4,711	91.07	3,396	90.51	873	92.68	442	92.28	
Yes	2,942	11.37	462	8.93	356	9.49	69	7.32	37	7.72	
Hypertension											<0.001
No	20,692	80.00	3,702	71.56	2,610	69.56	723	76.75	369	77.04	
Yes	5,173	20.00	1,471	28.44	1,142	30.44	219	23.25	110	22.96	
Hyperlipidemia											<0.001
No	21,917	84.74	4,229	81.75	3,049	81.26	799	84.82	381	79.54	
Yes	3,948	15.26	944	18.25	703	18.74	143	15.18	98	20.46	
Valvular heart disease											<0.001
No	24,995	96.64	4,915	95.01	3,577	95.34	881	93.52	457	95.41	
Yes	870	3.36	258	4.99	175	4.66	61	6.48	22	4.59	
Obesity											<0.001
No	25,603	98.99	5,085	98.30	3,695	98.48	921	97.77	469	97.91	
Yes	262	1.01	88	1.70	57	1.52	21	2.23	10	2.09	
COPD											<0.001
No	22,349	86.41	4,316	83.43	3,146	83.85	784	83.23	386	80.58	
Yes	3,516	13.59	857	16.57	606	16.15	158	16.77	93	19.42	
Liver disease											<0.001
No	20,653	79.85	3,930	75.97	2,857	76.15	721	76.54	352	73.49	
Yes	5,212	20.15	1,243	24.03	895	23.85	221	23.46	127	26.51	
Cancer											<0.001
No	24,675	95.40	5,016	96.97	3,637	96.93	913	96.92	466	97.29	
Yes	1,190	4.60	157	3.03	115	3.07	29	3.08	13	2.71	
Thyroid disease											<0.001
No	24,282	93.88	4,755	91.92	3,457	92.14	862	91.51	436	91.02	
Yes	1,583	6.12	418	8.08	295	7.86	80	8.49	43	8.98	
Autoimmune disease											0.793
No	25,805	99.77	5,160	99.75	3,742	99.73	942	100.00	476	99.37	
Yes	60	0.23	13	0.25	10	0.27	-	-	3	0.63	
Renal failure											0.016
No	25,578	98.89	5,135	99.27	3,723	99.23	935	99.26	477	99.58	
Yes	287	1.11	38	0.73	29	0.77	7	0.74	2	0.42	
Helicobacter pylori											0.014
No	25,790	99.71	5,147	99.50	3,737	99.60	935	99.26	475	99.16	
Yes	75	0.29	26	0.50	15	0.40	7	0.74	4	0.84	

a. Variables for propensity score matching. b. The difference between migraine patients and comparison after matching.

**Table 2:** Risk of related health outcomes in migraine patients with or without aura.

Variables	Peripheral artery disease						Stroke					
	Events	aHR ^a^	95% CI			p-value	Events	aHR ^a^	95% CI			p-value
Patients group												
Comparison (REF)	206	1					1,105	1				
Migraine	63	1.93	1.45	-	2.57	<0.001	268	1.55	1.35	-	1.77	<0.001
Migraine without aura	5	0.84	0.35	-	2.04	0.700	47	1.49	1.11	-	2.00	0.008
Migraine with aura	6	1.95	0.86	-	4.40	0.108	25	1.63	1.10	-	2.43	0.016
Gender												
Female (REF)	202	1					926	1				
Male	78	0.92	0.71	-	1.21	0.556	519	1.34	1.20	-	1.49	<0.001
Age (year)												
20-34 (REF)	15	1					52	1				
35-49	79	2.76	1.58	-	4.80	<0.001	287	3.04	2.26	-	4.08	<0.001
50-64	111	5.19	2.96	-	9.08	<0.001	623	9.47	7.09	-	12.65	<0.001
≥65	75	8.37	4.62	-	15.18	<0.001	483	17.81	13.19	-	24.06	<0.001
Diabetes mellitus												
No (REF)	214	1					1,083	1				
Yes	66	1.23	0.90	-	1.69	0.193	362	1.29	1.13	-	1.48	<0.001
Hypertension												
No (REF)	144	1					684	1				
Yes	136	1.65	1.24	-	2.19	<0.001	761	1.65	1.46	-	1.87	<0.001
Hyperlipidemia												
No (REF)	199	1					1,036	1				
Yes	81	1.16	0.86	-	1.56	0.336	409	0.98	0.86	-	1.12	0.788
Valvular heart disease												
No (REF)	267	1					1,348	1				
Yes	13	1.01	0.58	-	1.78	0.970	97	1.42	1.15	-	1.76	0.001
Obesity												
No (REF)	278	1					1,423	1				
Yes	2	0.55	0.14	-	2.23	0.406	22	1.33	0.87	-	2.03	0.190
COPD												
No (REF)	223	1					1,096	1				
Yes	57	1.01	0.75	-	1.37	0.947	349	1.12	0.99	-	1.27	0.080
Liver disease												
No (REF)	205	1					1,083	1				
Yes	75	1.12	0.85	-	1.48	0.421	362	0.95	0.84	-	1.07	0.389
Cancer												
No (REF)	265	1					1,346	1				
Yes	15	0.91	0.54	-	1.53	0.715	99	1.04	0.85	-	1.28	0.697
Thyroid disease												
No (REF)	261	1					1,359	1				
Yes	19	1.05	0.65	-	1.68	0.853	86	0.95	0.76	-	1.19	0.650
Autoimmune disease												
No (REF)	279	1					1,442	1				
Yes	1	1.98	0.28	-	14.13	0.498	3	0.92	0.23	-	3.67	0.901
Renal failure												
No (REF)	268	1					1,409	1				
Yes	12	2.49	1.38	-	4.51	0.003	36	1.19	0.85	-	1.67	0.300
Helicobacter pylori												
No (REF)	280						1,444	1				
Yes	-	-		-		-	1	0.27	0.04	-	1.92	0.191

a. aHR, adjusted hazard ratio.

## Abbreviations

aHR: adjusted hazard ratio
CI: confidence interval
PAD: peripheral artery disease
ABI: ankle-brachial index
EMP: endothelial microparticles
*H. pylori*: Helicobacter pylori
COVID-19: coronavirus disease 2019

## References

[1] Feigin V (2016). Global, regional, and National Incidence, prevalence, and years lived with disability for 310 acute and chronic diseases and injuries, 1990-2015: a systematic analysis for the global burden of disease study 2015. The Lancet.

[2] Wang SJ, Fuh JL, Young YH, Lu SR, Shia BC (2000). Prevalence of migraine in Taipei, Taiwan: a population-based survey. Cephalalgia.

[3] Saeed A, Rana KF, Warriach ZI, Tariq MA, Malik BH (2019). Association of Migraine and Ischemic Heart Disease: A Review. Cureus.

[4] Sacco S, Ornello R, Ripa P, Tiseo C, Degan D, Pistoia F (2015). Migraine and risk of ischaemic heart disease: a systematic review and meta-analysis of observational studies. European journal of neurology.

[5] Mahmoud AN, Mentias A, Elgendy AY, Qazi A, Barakat AF, Saad M (2018). Migraine and the risk of cardiovascular and cerebrovascular events: a meta-analysis of 16 cohort studies including 1 152 407 subjects. BMJ open.

[6] Norman PE, Eikelboom JW, Hankey GJ (2004). Peripheral arterial disease: prognostic significance and prevention of atherothrombotic complications. Medical Journal of Australia.

[7] Bergiers S, Vaes B, Degryse J (2011). To screen or not to screen for peripheral arterial disease in subjects aged 80 and over in primary health care: a cross-sectional analysis from the BELFRAIL study. BMC family practice.

[8] Hirsch AT, Haskal ZJ, Hertzer NR, Bakal CW, Creager MA, Halperin JL (2006). ACC/AHA 2005 guidelines for the management of patients with peripheral arterial disease (lower extremity, renal, mesenteric, and abdominal aortic): a collaborative report from the American Association for Vascular Surgery/Society for Vascular Surgery, Society for Cardiovascular Angiography and Interventions, Society for Vascular Medicine and Biology, Society of Interventional Radiology, and the ACC/AHA Task Force on Practice Guidelines (Writing Committee to Develop Guidelines for the Management of Patients With Peripheral Arterial Disease). Journal of the American College of Cardiology.

[9] Jurno ME, Chevtchouk L, Nunes AA, De Rezende DF, da Cunha Jevoux C, De Souza JA (2010). Ankle-brachial index, a screening for peripheral obstructive arterial disease, and migraine-a controlled study. Headache: The Journal of Head and Face Pain.

[10] Bigal M, Kurth T, Santanello N, Buse D, Golden W, Robbins M (2010). Migraine and cardiovascular disease: a population-based study. Neurology.

[11] Adelborg K, Szépligeti SK, Holland-Bill L, Ehrenstein V, Horváth-Puhó E, Henderson VW (2018). Migraine and risk of cardiovascular diseases: Danish population based matched cohort study. bmj.

[12] Lee T-Y, Hsu Y-C, Tseng H-C, Yu S-H, Lin J-T, Wu M-S (2019). Association of daily aspirin therapy with risk of hepatocellular carcinoma in patients with chronic hepatitis B. JAMA internal medicine.

[13] Cheng Y-T, Cheng C-T, Wang S-Y, Wu VC-C, Chu P-H, Chou A-H (2019). Long-term outcomes of endovascular and open repair for traumatic thoracic aortic injury. JAMA network open.

[14] Schürks M, Rist PM, Bigal ME, Buring JE, Lipton RB, Kurth T (2009). Migraine and cardiovascular disease: systematic review and meta-analysis. Bmj.

[15] Sacco S, Ripa P, Grassi D, Pistoia F, Ornello R, Carolei A (2013). Peripheral vascular dysfunction in migraine: a review. The journal of headache and pain.

[16] Liman TG, Bachelier-Walenta K, Neeb L, Rosinski J, Reuter U, Böhm M (2015). Circulating endothelial microparticles in female migraineurs with aura. Cephalalgia.

[17] Tietjen GE, Khubchandani J, Herial N, Palm-Meinders IH, Koppen H, Terwindt GM (2018). Migraine and vascular disease biomarkers: a population-based case-control study. Cephalalgia.

[18] Winsvold BS, Bettella F, Witoelar A, Anttila V, Gormley P, Kurth T (2017). Shared genetic risk between migraine and coronary artery disease: a genome-wide analysis of common variants. PloS one.

[19] Kullo IJ, Leeper NJ (2015). The genetic basis of peripheral arterial disease: current knowledge, challenges, and future directions. Circulation research.

[20] Selvin E, Erlinger TP (2004). Prevalence of and risk factors for peripheral arterial disease in the United States: results from the National Health and Nutrition Examination Survey, 1999-2000. Circulation.

[21] Ludhwani D, Wu J (2019). Obesity paradox in peripheral arterial disease: results of a propensity match analysis from the national inpatient sample. Cureus.

[22] Stam AH, Weller CM, Janssens ACJ, Aulchenko YS, Oostra BA, Frants RR Migraine is not associated with enhanced atherosclerosis. 2013; 33: 228-35.

[23] Kuo F-H, Lee C-Y, Li J-P, Chung J-F, Wang Y-H, Hsieh M-J (2020). Migraine as a Risk Factor for Peripheral Artery Occlusive Disease: A Population-Based Cohort Study. International Journal of Environmental Research and Public Health.

[24] Brevetti G, Giugliano G, Brevetti L, Hiatt WR (2010). Inflammation in peripheral artery disease. Circulation.

[25] Liang KP, Liang KV, Matteson EL, McClelland RL, Christianson TJ, Turesson C (2006). Incidence of noncardiac vascular disease in rheumatoid arthritis and relationship to extraarticular disease manifestations. Arthritis & Rheumatism: Official Journal of the American College of Rheumatology.

[26] Bloemenkamp DG, van den Bosch MA, Mali WP, Tanis BC, Rosendaal FR, Kemmeren JM (2002). Novel risk factors for peripheral arterial disease in young women. The American journal of medicine.

[27] Bloemenkamp DG, Willem PTM, Tanis BC, Rosendaal FR, van den Bosch MA, Kemmeren JM (2002). Chlamydia pneumoniae, Helicobacter pylori and cytomegalovirus infections and the risk of peripheral arterial disease in young women. Atherosclerosis.

[28] Gasbarrini A, Franceschi F, Armuzzi A, Ojetti V, Candelli M, Torre ES (1999). Extradigestive manifestations of Helicobacter pylori gastric infection. Gut.

[29] Sawayama Y, Hamada M, Otaguro S, Maeda S, Ohnishi H, Fujimoto Y (2008). Chronic Helicobacter pylori infection is associated with peripheral arterial disease. Journal of Infection and Chemotherapy.

[30] Gasbarrini A, De AL, Fiore G, Gambrielli M, Franceschi F, Ojetti V (1998). Beneficial effects of Helicobacter pylori eradication on migraine. Hepato-gastroenterology.

[31] Gasbarrini A, Serricchio M, Tondi P, De AL, Franceschi F, Ojetti V (1998). Raynaud's Phenomenon and Helicobacter Pylori Infection. The International journal of angiology: official publication of the International College of Angiology, Inc.

[32] Toptan T, Aktan Ç, Başarı A, Bolay H (2020). Case Series of Headache Characteristics in COVID-19: Headache Can Be an Isolated Symptom. Headache: The Journal of Head and Face Pain.

[33] Ogawa M, Doo FX, Somwaru AS, Roudenko A, Kamath A, Friedman B (2021). Peripheral arterial occlusion due to COVID-19: CT angiography findings of nine patients. Clinical Imaging.

[34] Bellosta R, Luzzani L, Natalini G, Pegorer MA, Attisani L, Cossu LG (2020). Acute limb ischemia in patients with COVID-19 pneumonia. Journal of vascular surgery.

[35] Teodorescu VJ, Vavra AK, Kibbe MR (2013). Peripheral arterial disease in women. Journal of vascular surgery.

[36] Bush RL, Kallen MA, Liles DR, Bates JT, Petersen LA (2008). Knowledge and awareness of peripheral vascular disease are poor among women at risk for cardiovascular disease. Journal of Surgical Research.

