# Supplementary material for: Risk of peripheral artery disease and stroke in migraineurs with or without aura: a nationwide population-based cohort study
Source: Int J Med Sci. 2022 Jul 4;19(7):1163–72. doi: 10.7150/ijms.72119 (PMC9339415; doi:10.7150/ijms.72119)
Supplement: Supplementary file 1 — Supplementary table. [file ijmsv19p1163s1.pdf]

Table S1. The variables used for propensity score matching

Variables	Definitions	ICD-9-CM[b]
Age (year)	20-34<br>35-49<br>50-64<br>≥65	
Gender	Female<br>Male	
The year of enrollment		
	2002	
	2003	
	2004	
	2005	
	2006	
	2007	
	2008	
	2009	
	2010	
	2011	
CCI[a]		
	Myocardial infarction	410.x, 412.x
	Congestive heart failure	398.91, 402.01, 402.11, 402.91,<br>404.01, 404.03, 404.11, 404.13, 404.91, 404.93, 425.4–425.9, 428.x
	Peripheral vascular disease	093.0, 437.3, 440.x, 441.x, 443.1–443.9, 47.1, 557.1, 557.9, V43.4
	Cerebrovascular disease	362.34, 430.x–438.x
	Dementia	290.x, 294.1, 331.2
	Chronic obstructive disease	416.8, 416.9, 490.x–505.x, 506.4, 508.1, 508.8
	Connective tissue disease	446.5, 710.0–710.4, 714.0–714.2, 714.8, 725.x
	Peptic ulcer disease	531.x–534.x
	Liver disease	070.22, 070.23, 070.32, 070.33,

		070.44, 070.54, 070.6, 070.9, 570.x, 571.x, 573.3, 573.4, 573.8, 573.9, V42.7<br>456.0–456.2, 572.2–572.8
	Diabetes mellitus	250.0–250.3, 250.8, 250.9<br>250.4–250.7
	Hemiplegia or paraplegia	334.1, 342.x, 343.x, 344.0–344.6, 344.9
	Moderate to severe chronic kidney disease	403.01, 403.11, 403.91, 404.02,<br>404.03, 404.12, 404.13, 404.92, 404.93, 582.x, 583.0–583.7, 585.x, 586.x, 588.0, V42.0, V45.1, V56.x
	Solid tumor	196.x–199.x
	Leukemia/ lymphoma	140.x–172.x, 174.x–195.8, 200.x–208.x, 238.6
	Acquired immunodeficiency syndrome	042.x–044.x

[a] Charlson comorbidities index

[b] International Classification of Diseases, Ninth Revision, Clinical Modification
